# Complete Mitochondrial Genome of *Ovophis makazayazaya* (Viperidae, Crotalinae) and Phylogenetic Analysis

**DOI:** 10.1002/ece3.73747

**Published:** 2026-05-27

**Authors:** Yuheng Jiang, Yuhao Nie, Zhiqiang Ge

**Affiliations:** ^1^ College of Life Science and Technology Xinjiang University Ürümqi China; ^2^ International Sakharov Environmental Institute Belarusian State University Minsk Republic of Belarus; ^3^ Belarus–Russia–Tajikistan Joint Laboratory for Ecology and Environmental Research Lomonosov Moscow State University Dushanbe Republic of Tajikistan

**Keywords:** Crotalinae, mitogenome, *Ovophis makazayazaya*, phylogeny, protein‐coding genes

## Abstract

This research sequenced and annotated the complete mitochondrial genome of *Ovophis makazayazaya* and assessed its phylogenetic placement within Crotalinae. The complete mitochondrial genome of *O. makazayazaya* was determined to be 17,230 bp in length, comprising 13 protein‐coding genes (PCGs), 2 rRNA genes, 22 tRNA genes, and 1 mitochondrial control region (D‐loop). The base composition of the mitochondrial genome of *O. makazayazaya* is as follows: A (32.0%), T (25.2%), C (29.7%), and G (13.1%). The nucleotide composition exhibited a significant AT bias (57.2%). Among the 13 protein‐coding genes, only ATG, ATT, GTG, and ATC serve as the initiation codons of protein‐coding sequences. The termination codons are mainly TAA, AGA, and AGG; while ND1, ND2, COX2, COX3, ND3, and CYTB terminate with incomplete stop codons (e.g., T‐‐). All 22 tRNAs formed the canonical cloverleaf structure except tRNA‐Ser(AGY), which lacked the DHU arm. All PCGs had Ka/Ks < 1, consistent with purifying selection; p‐distance values were < 1 across genes. Phylogenetic analyses based on the concatenated sequences of 13 mitochondrial protein‐coding genes recovered *O. makazayazaya* within Crotalinae in both Bayesian inference and maximum‐likelihood trees. In the present mitochondrial phylogeny, *O. makazayazaya* was recovered close to *Protobothrops*, supporting its phylogenetic placement among Asian crotaline pitvipers. Because only one *Ovophis* species was included in the present mitogenomic dataset, this result should be interpreted as evidence for the placement of *O. makazayazaya* within Crotalinae rather than as a direct test of *Ovophis* monophyly. Together, the mitogenomic features and phylogenetic results provide a useful reference for comparative analyses of crotaline systematics, mitochondrial genome evolution, and future phylogenetic studies of Asian pitvipers.

## Graphical abstract

This study sequenced and annotated the complete 17,230 bp mitochondrial genome of *Ovophis makazayazaya* , which contains 13 protein‐coding genes (PCGs), 2 rRNAs, 22 tRNAs, and a D‐loop region, with a significant AT bias (57.2%). Phylogenetic analyses confirmed its placement within Crotalinae, and all PCGs showed evidence of purifying selection (Ka/Ks < 1), providing a valuable reference for comparative studies of crotaline mitogenome evolution.

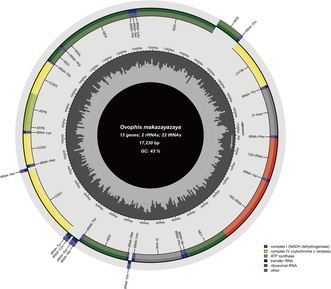

## 1 Introduction

**FIGURE 1:**
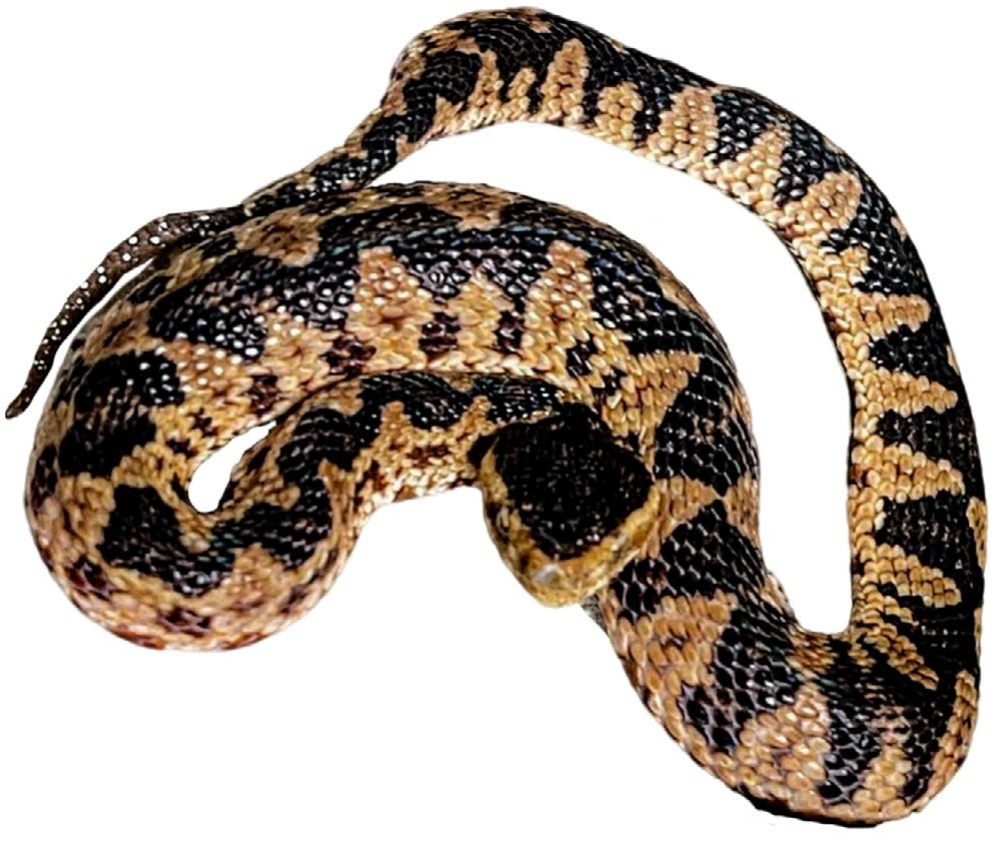
*Ovophis makazayazaya* .

The genus *Ovophis* was first proposed in Burger's doctoral dissertation (Burger 1971) and was subsequently adopted in later taxonomic syntheses of crotaline pitvipers (Hoge and Romano‐Hoge 1981). The Taiwanese mountain pitviper, a member of Viperidae (subfamily Crotalinae), was originally described from material collected in Pingtung, Taiwan, as *Trimeresurus makazayazaya* (Takahashi 1922). It was later treated for decades as a subspecies within the *Trimeresurus monticola* complex under the name *Trimeresurus monticola makazayazaya* (later *Ovophis monticola makazayazaya* ), and was considered endemic to Taiwan (Takahashi 1930). Using phylogenetic reconstruction based on four mitochondrial gene fragments together with comparative analyses of external morphology, this taxon was reinstated as a distinct species, *Ovophis makazayazaya* (Figure 1), and its documented distribution was expanded to include Sichuan and Yunnan in China as well as northern Vietnam (Malhotra et al. 2011). More recently, integrative comparisons of morphology and multilocus genetic data from multiple localities in China supported the restriction of its sister lineage ( *Ovophis monticola* sensu stricto) to Xizang (Tibet), while indicating that the distribution of *O. makazayazaya* in China is broadly consistent with the range proposed previously (Zeng et al. 2023). The species is currently assessed as Least Concern on the IUCN Red List (Lau et al. 2012), but it is included in China's national inventory of terrestrial wildlife under state protection that are considered beneficial or of important economic or scientific research value (State Forestry Administration of China 2000).

Despite increasing molecular work on *Ovophis*, complete mitochondrial genomes remain limited for this group. A complete mitogenome has been reported for *Ovophis okinavensis* (Dong and Kumazawa 2005), whereas mitogenomic resources for other species remain scarce. Previous phylogenetic studies based on mitochondrial fragments and multilocus datasets have clarified several taxonomic issues within *Ovophis* and related Asian pitvipers, but they also indicate that broader genomic sampling is still needed to improve the resolution of species relationships and evolutionary history in this group (Malhotra et al. 2011; Zeng et al. 2023). In this study, we sequenced and annotated the complete mitochondrial genome of *O. makazayazaya* using high‐throughput sequencing, characterized its nucleotide composition, gene arrangement, codon usage, RNA genes, and selective constraints, and reconstructed its phylogenetic placement within Crotalinae based on 13 mitochondrial protein‐coding genes. These data provide a new genomic resource for comparative mitogenomic analyses and future phylogenetic studies of Asian pitvipers.

## 2 Materials and Methods

### 2.1 Sample Collection

A road‐killed adult specimen of *O. makazayazaya* was collected from Yangshan River Scenic Area, Dejiang County, Guizhou Province, China (28°26′0.050″ N, 108°8′1.581″ E). Sample collection was performed in accordance with the laws and regulations of the People's Republic of China. Because the specimen was found dead on the road and no live animal was captured or sacrificed for this study, no additional ethical approval was required. Muscle tissue was preserved in 99.9% ethanol and stored at −20°C until DNA extraction.

### 2.2 Laboratory Procedures

#### 2.2.1 Genomic DNA Extraction and Species Verification

Genomic DNA was extracted from ethanol‐preserved muscle using the TIANamp Genomic DNA Kit (TIANGEN, Beijing, China) following the manufacturer's protocol and stored at −20°C. Although the complete mitochondrial genome contains the COI gene, an independent COI fragment was amplified and Sanger‐sequenced before mitogenome assembly to verify the taxonomic identity of the specimen. This step served as an independent molecular confirmation of the morphology‐based identification and reduced the risk of using a misidentified specimen for subsequent high‐throughput sequencing and mitochondrial genome annotation. To confirm the morphological identification, a fragment of the mitochondrial cytochrome c oxidase subunit I gene (COI) was amplified using primers VF1‐t1 (5′‐TGTAAAACGACGGCCAGTTCTCAACCAACCACAAAGACATTGG‐3′) and VR1‐t1 (5′‐CAGGAAACAGCTATGACTTCTGGGTGGCCAAAGAATCA‐3′). PCR amplifications were performed in a 20 μL reaction volume containing 10 μL of 2 × F8 LongFast PCR MasterMix, 0.5 μL of each primer (10 μM), 1 μL of template DNA, and 8 μL of sterile distilled water. The thermal cycling conditions were as follows: initial denaturation at 95°C for 10 min; 30 cycles of denaturation at 95°C for 30 s, annealing at 58°C for 30 s, and extension at 72°C for 30 s; and a final extension at 72°C for 10 min. PCR products were Sanger‐sequenced (Sangon Biotech, Shanghai, China), and the resulting sequence was queried against the NCBI nucleotide database using BLAST. The best matches supported assignment to *O. makazayazaya* .

#### 2.2.2 Mitochondrial Genome Sequencing, Assembly, and Annotation

A subset of muscle tissue from the same individual was used for high‐throughput sequencing by Berry Genomics (Beijing, China). A paired‐end genomic DNA library with an insert size of approximately 350 bp was constructed and sequenced on an Illumina NovaSeq 6000 platform, generating paired‐end 250 bp reads in FASTQ format. The total data yield was approximately 10 Gb, corresponding to an approximate 5× depth at the nuclear‐genome scale, as reported by the sequencing provider. Raw reads were quality‐filtered using fastp v0.19.7 (Chen et al. 2018) to remove adapter contamination, low‐quality reads, highly duplicated reads, and reads containing excessive ambiguous bases.

The mitochondrial genome was assembled in Geneious Prime 2024 (Biomatters Ltd., Auckland, New Zealand; Kearse et al. 2012) using a reference‐guided “Map to Reference” approach, with the complete mitochondrial genome of *Ovophis okinavensis* (NC_007397.1) used as the reference sequence. Protein‐coding genes were initially identified using NCBI ORFfinder (Sayers et al. 2023) and annotated by similarity searches against the NCBI nucleotide database using BLAST (Altschul et al. 1990). Transfer RNA genes and their putative secondary structures were predicted using tRNAscan‐SE v2.0 (Chan and Lowe 2019). The complete annotation was further inspected and refined using the MITOS web server (Bernt et al. 2013). Sequence alignments used in downstream analyses were performed in MEGA 11 (Tamura et al. 2021). The circular mitochondrial genome map was generated using the MITOS visualization platform and manually checked against the final annotation.

### 2.3 Sequence Analyses

Base composition was calculated for the complete mitogenome and for major genomic regions using MEGA 11 (Tamura et al. 2021). Nucleotide skews were computed using the formulas AT‐skew = (A − T)/(A + T) and GC‐skew = (G − C)/(G + C). Relative synonymous codon usage (RSCU) values were calculated for the 13 mitochondrial protein‐coding genes in MEGA 11, and codon usage plots were generated using PhyloSuite v1.2.3 (Zhang et al. 2020).

Pairwise genetic distances for each protein‐coding gene were calculated among the sampled crotaline species using the p‐distance model in MEGA 11. Mean p‐distance values were used to compare relative nucleotide divergence among mitochondrial protein‐coding genes. Evolutionary rates of protein‐coding genes were assessed using the ratio of nonsynonymous to synonymous substitutions (Ka/Ks) in DnaSP v6 (Rozas et al. 2017). Ka/Ks values below 1 were interpreted as evidence of purifying selection, whereas higher values indicated relatively relaxed selective constraints among genes.

### 2.4 Phylogenetic Reconstruction

Phylogenetic reconstruction was performed using Bayesian inference and maximum likelihood methods based on the concatenated nucleotide sequences of 13 mitochondrial protein‐coding genes from 33 Crotalinae species. *Daboia russelii* (NC_011391.1), a representative of Viperinae, was selected as the outgroup. GenBank sequences were retrieved from the NCBI nucleotide database and managed in PhyloSuite v1.2.3 (Zhang et al. 2020). Individual protein‐coding genes were aligned using MAFFT (Katoh and Standley 2013), checked and optimized using MACSE (Ranwez et al. 2018), and ambiguously aligned regions were removed using Gblocks (Talavera and Castresana 2007). The aligned protein‐coding genes were then concatenated into a single matrix.

The best‐fit partitioning schemes and nucleotide substitution models were selected using ModelFinder (Kalyaanamoorthy et al. 2017) according to the Bayesian Information Criterion. Maximum‐likelihood analysis was conducted using IQ‐TREE (Nguyen et al. 2015), and nodal support was evaluated using 1000 ultrafast bootstrap replicates. Bayesian inference was performed using MrBayes (Ronquist et al. 2012), with two independent runs and four Markov chains. The first 25% of sampled trees were discarded as burn‐in, and the remaining trees were used to generate a majority‐rule consensus tree. The final phylogenetic trees were visualized and edited using iTOL v6 (Letunic and Bork 2024).

## 3 Results

### 3.1 Base Composition and Nucleotide Skew

**FIGURE 2:**
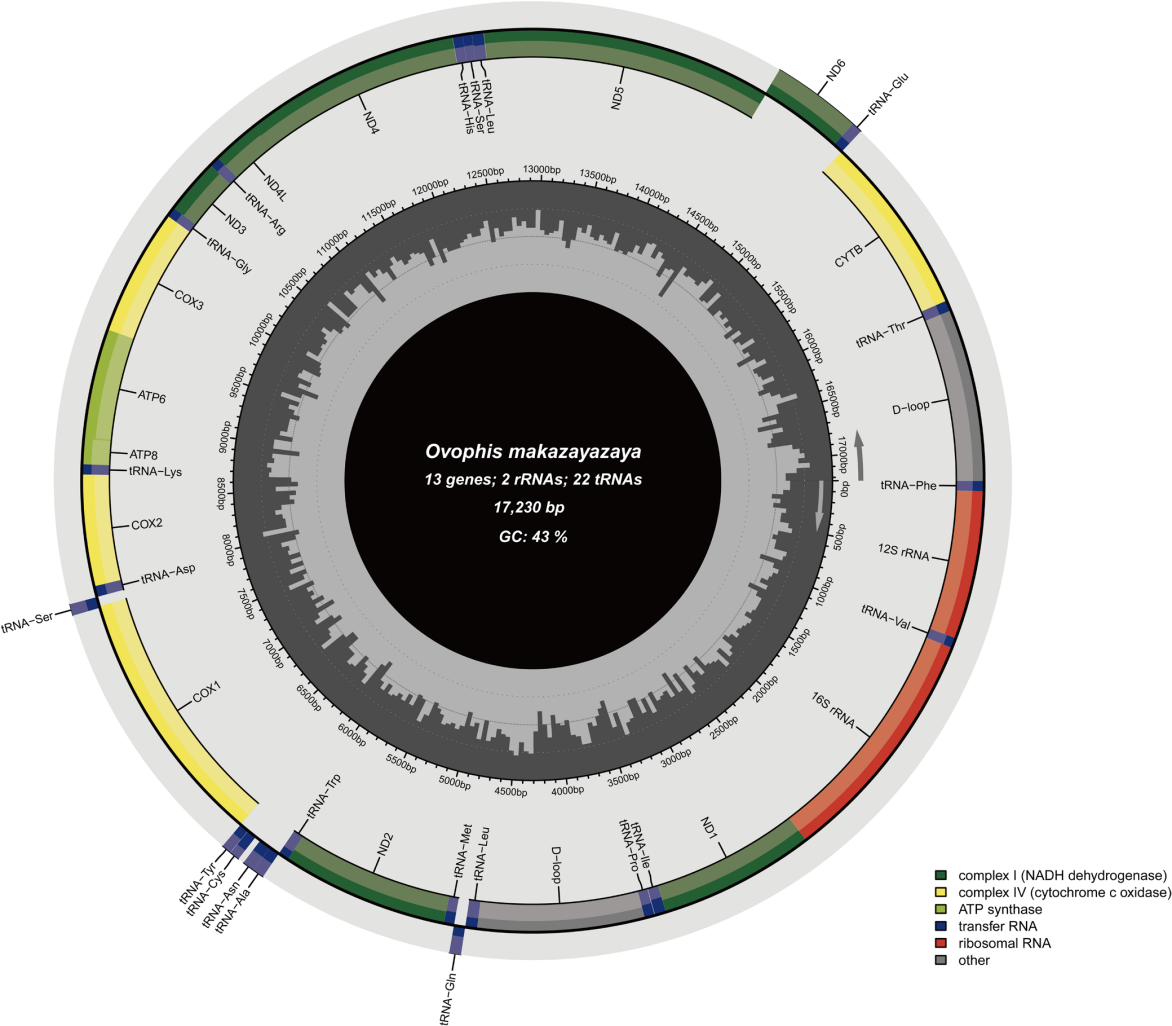
Map of the *O. makazayazaya* mitochondrial genome.

The complete mitochondrial genome of *O. makazayazaya* was 17,230 bp in length and contained 13 protein‐coding genes, 22 tRNA genes, two rRNA genes, and one control region (Tables 1 and 2; Figure 2). Across the whole mitogenome, the nucleotide composition was A = 32.0%, T = 25.2%, C = 29.7%, and G = 13.1% (Table 3). The genome showed a clear A + T enrichment, with an A + T content of 57.2% and a G + C content of 42.8%.

**TABLE 1:** Basic information for the species analyzed in this study.

No.	Subfamily	Genus	Species	Accession	Length (bp)	AT%
1	Crotalinae	*Agkistrodon*	*Agkistrodon piscivorus*	NC_009768.1	17,213	57.7
2	Crotalinae	*Agkistrodon*	*Agkistrodon contortrix*	NC_035638.1	16,269	57.5
3	Crotalinae	*Bothrops*	*Bothrops diporus*	NC_039649.1	17,642	56.1
4	Crotalinae	*Bothrops*	*Bothrops pubescens*	NC_039648.1	17,694	56.1
5	Crotalinae	*Bothrops*	*Bothrops jararaca*	NC_030760.1	17,526	56.4
6	Crotalinae	*Crotalus*	*Crotalus adamanteus*	NC_041524.1	17,242	57
7	Crotalinae	*Crotalus*	*Crotalus horridus*	HM641837.1	17,260	57.1
8	Crotalinae	*Deinagkistrodon*	*Deinagkistrodon acutus*	NC_010223.1	17,548	57.8
9	Crotalinae	*Ovophis*	*Ovophis makazayazaya*	PV608488	17,230	57.2
10	Crotalinae	*Gloydius*	*Gloydius intermedius*	NC_025560.1	17,226	58.3
11	Crotalinae	*Gloydius*	*Gloydius shedaoensis*	NC_029424.1	17,222	58.2
12	Crotalinae	*Gloydius*	*Gloydius rubromaculatus*	NC_064056.1	17,224	58
13	Crotalinae	*Gloydius*	*Gloydius himalayanus*	NC_068353.1	17,210	57.5
14	Crotalinae	*Gloydius*	*Gloydius strauchi*	NC_036234.1	17,224	57.9
15	Crotalinae	*Gloydius*	*Gloydius ussuriensis*	NC_026553.1	17,208	58.7
16	Crotalinae	*Gloydius*	*Gloydius blomhoffi*	NC_011390.1	17,227	58.1
17	Crotalinae	*Gloydius*	*Gloydius changdaoensis*	MT731652.1	17,224	58.3
18	Crotalinae	*Lachesis*	*Lachesis muta*	NC_081003.1	17,177	59.3
19	Crotalinae	*Protobothrops*	*Protobothrops flavoviridis*	NC_030181.1	17,232	58.1
20	Crotalinae	*Protobothrops*	*Protobothrops tokarensis*	NC_030182.1	17,233	58.2
21	Crotalinae	*Protobothrops*	*Protobothrops kaulbacki*	NC_029166.1	17,237	57.9
22	Crotalinae	*Protobothrops*	*Protobothrops mangshanensis*	NC_026052.1	17,230	56.5
23	Crotalinae	*Protobothrops*	*Protobothrops mucrosquamatus*	NC_021412.1	17,234	58.2
24	Crotalinae	*Protobothrops*	*Protobothrops himalayanus*	NC_029165.1	17,389	57.7
25	Crotalinae	*Protobothrops*	*Protobothrops dabieshanensis*	NC_022473.1	17,193	58.3
26	Crotalinae	*Protobothrops*	*Protobothrops cornutus*	NC_022695.1	17,219	58
27	Crotalinae	*Protobothrops*	*Protobothrops maolanensis*	NC_026051.1	17,228	57.9
28	Crotalinae	*Protobothrops*	*Protobothrops jerdonii*	NC_021402.1	17,239	58.2
29	Crotalinae	*Protobothrops*	*Protobothrops xiangchengensis*	KF460436.1	17,240	58.4
30	Crotalinae	*Sistrurus*	*Sistrurus catenatus*	NC_071935.1	17,245	58.3
31	Crotalinae	*Sistrurus*	*Sistrurus miliarius*	MK330875.1	17,777	59.4
32	Crotalinae	*Trimeresurus*	*Trimeresurus albolabris*	NC_022820.1	17,220	60.4
33	Crotalinae	*Trimeresurus*	*Trimeresurus sichuanensis*	NC_029494.1	17,225	59.2

*Note:* Accession numbers correspond to complete mitochondrial genomes retrieved from GenBank. AT% indicates the A + T content of the complete mitochondrial genome.

**TABLE 2:** Annotation of the *O. makazayazaya* mitochondrial genome.

Gene	Position		Size	Intergenic nucleotides	Codon		Strand
	From	To			Start	Stop	
tRNA‐Phe	1	64	64				H
12S rRNA	65	977	913				H
tRNA‐Val	978	1040	63				H
16S rRNA	1041	2525	1485				H
ND1	2526	3486	961		ATG	T	H
tRNA‐Ile	3487	3555	69				H
tRNA‐Pro	3558	3620	63	2			L
tRNA‐Leu1	4648	4720	73	1027			H
tRNA‐Gln	4721	4789	69				L
tRNA‐Met	4790	4852	63				H
ND2	4853	5882	1030		ATT	T	H
tRNA‐Trp	5883	5948	66				H
tRNA‐Ala	5949	6013	65				L
tRNA‐Asn	6014	6086	73				L
tRNA‐Cys	6122	6180	59	35			L
tRNA‐Tyr	6182	6239	58	1			L
COX1	6241	7842	1602	1	GTG	AGA	H
tRNA‐Ser1	7833	7900	68	−10			L
tRNA‐Asp	7901	7967	67				H
COX2	7968	8652	685		ATG	T	H
tRNA‐Lys	8653	8715	63				H
ATP8	8716	8880	165		ATG	TAA	H
ATP6	8871	9551	681	−10	ATG	TAA	H
COX3	9551	10,334	784	−1	ATG	T	H
tRNA‐Gly	10,335	10,395	61				H
ND3	10,396	10,738	343		ATC	T	H
tRNA‐Arg	10,739	10,801	63				H
ND4L	10,802	11,092	291		ATG	TAA	H
ND4	11,092	12,429	1338	−1	ATG	AGA	H
tRNA‐His	12,431	12,492	62	1			H
tRNA‐Ser2	12,493	12,549	57				H
tRNA‐Leu2	12,550	12,621	72				H
ND5	12,623	14,410	1788	1	ATG	TAA	H
ND6	14,406	14,927	522	−5	ATG	AGG	L
tRNA‐Glu	14,928	14,990	63				L
CYTB	14,991	16,104	1114		ATG	T	H
tRNA‐Thr	16,105	16,170	66				H
D‐loop	16,171	17,230	1060	0			

*Note:* H and L indicate genes encoded on the heavy and light strands of the mitochondrial genome, respectively. Positive values in the “intergenic nucleotides” column indicate the number of nucleotides between adjacent genes, whereas negative values indicate overlapping nucleotides. The 1027 bp region between tRNA‐Pro and tRNA‐Leu1 represents a large non‐coding intergenic spacer identified in the mitogenome annotation.

**TABLE 3:** Base composition and skewness of the *O. makazayazaya* mitogenome.

Region	Length (bp)	A%	C%	G%	T%	G + C%	A + T%	GC‐skew	AT‐skew
Total genome	17,230	32.0	29.7	13.1	25.2	42.8	57.2	−0.388	0.119
PCGs	11,304	31.8	31.8	11.9	24.6	43.7	56.4	−0.455	0.128
tRNAs	1427	33.3	24.7	17	25	41.7	58.3	−0.185	0.142
rRNAs	2398	37.2	24.8	16.8	21.2	41.6	58.4	−0.192	0.274
D‐loop	1060	27	27.5	12.5	33.1	39.9	60.1	−0.375	−0.101

Nucleotide skew analyses further supported this compositional bias. The whole mitogenome exhibited a positive AT‐skew of 0.119 and a negative GC‐skew of −0.388, indicating an excess of A over T and an excess of C over G. Similar trends were observed in the protein‐coding genes, tRNAs, and rRNAs, although the D‐loop showed a slightly negative AT‐skew (Table 3). A large non‐coding intergenic spacer of 1027 bp was detected between tRNA‐Pro and tRNA‐Leu1. This region was retained in the annotation because no additional protein‐coding gene or tRNA gene was predicted within this interval.

### 3.2 Protein‐Coding Genes and Codon Usage Patterns

The 13 mitochondrial protein‐coding genes of *O. makazayazaya* totaled 11,304 bp, accounting for 65.6% of the complete mitogenome (Table 2). Most protein‐coding genes initiated with the canonical ATG start codon, including ND1, COX2, ATP8, ATP6, COX3, ND4, ND4L, ND5, ND6, and CYTB. Alternative initiation codons were detected in three genes: ND2 used ATT, COX1 used GTG, and ND3 used ATC (Table 2).

Stop codon usage varied among genes. Four protein‐coding genes, ATP8, ATP6, ND4L, and ND5, terminated with TAA. COX1 and ND4 ended with AGA, and ND6 ended with AGG. The remaining six genes, ND1, ND2, COX2, COX3, ND3, and CYTB, had incomplete T stop codons, which are typically completed by post‐transcriptional polyadenylation (Table 2).

**FIGURE 3:**
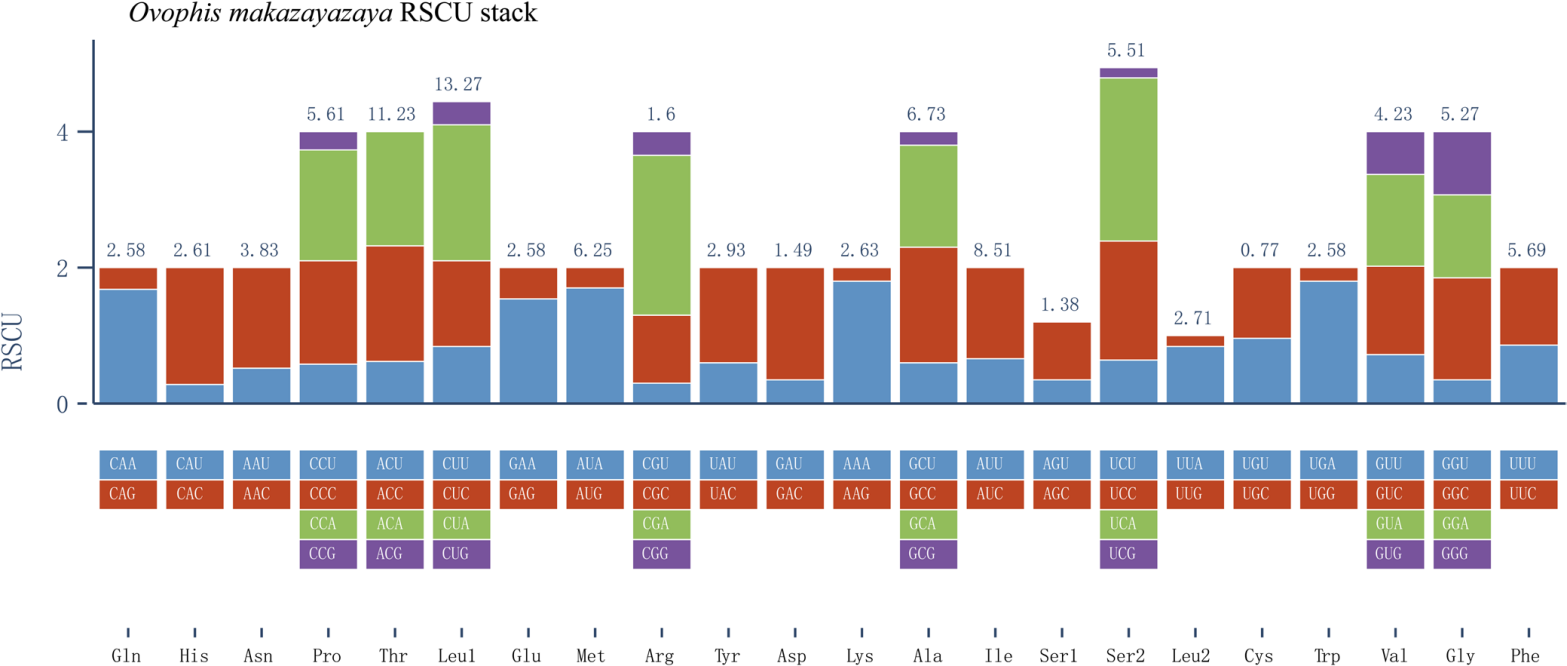
Relative synonymous codon usage of the 13 mitochondrial protein‐coding genes in *O. makazayazaya*. (The numbers above the bars indicate the observed counts of each codon in the concatenated protein‐coding gene dataset.)

Codon usage bias was assessed using relative synonymous codon usage (RSCU) (Figure 3; Table 4). The codon CUA showed the highest RSCU value (2.44), whereas ACG showed the lowest RSCU value (0.11), indicating heterogeneous synonymous codon preferences among mitochondrial protein‐coding genes.

**TABLE 4:** Relative synonymous codon usage (RSCU) in the mitochondrial protein‐coding genes of *O. makazayazaya* .

Amino acid	Codon	*n*	RSCU	Amino acid	Codon	*n*	RSCU
Phe	UUU	93	0.87	Ala	GCA	94	1.49
Phe	UUC	121	1.13	Ala	GCG	13	0.21
Leu2	UUA	86	0.86	Tyr	UAU	35	0.64
Leu2	UUG	16	0.16	Tyr	UAC	75	1.36
Leu1	CUU	84	0.84	His	CAU	15	0.31
Leu1	CUC	126	1.26	His	CAC	83	1.69
Leu1	CUA	244	2.44	Gln	CAA	81	1.67
Leu1	CUG	45	0.45	Gln	CAG	16	0.33
Ile	AUU	105	0.66	Asn	AAU	41	0.57
Ile	AUC	215	1.34	Asn	AAC	103	1.43
Met	AUA	195	1.66	Lys	AAA	89	1.8
Met	AUG	40	0.34	Lys	AAG	10	0.2
Val	GUU	29	0.73	Asp	GAU	11	0.39
Val	GUC	52	1.31	Asp	GAC	45	1.61
Val	GUA	53	1.33	Glu	GAA	73	1.51
Val	GUG	25	0.63	Glu	GAG	24	0.49
Ser2	UCU	29	0.67	Cys	UGU	14	0.97
Ser2	UCC	76	1.76	Cys	UGC	15	1.03
Ser2	UCA	96	2.22	Trp	UGA	87	1.79
Ser2	UCG	6	0.14	Trp	UGG	10	0.21
Pro	CCU	32	0.61	Arg	CGU	5	0.33
Pro	CCC	79	1.5	Arg	CGC	15	1
Pro	CCA	87	1.65	Arg	CGA	35	2.33
Pro	CCG	13	0.25	Arg	CGG	5	0.33
Thr	ACU	69	0.65	Ser1	AGU	16	0.37
Thr	ACC	175	1.66	Ser1	AGC	36	0.83
Thr	ACA	166	1.57	Gly	GGU	19	0.38
Thr	ACG	12	0.11	Gly	GGC	74	1.49
Ala	GCU	39	0.62	Gly	GGA	61	1.23
Ala	GCC	107	1.69	Gly	GGG	44	0.89

### 3.3 Genetic Divergence and Selective Constraints (p‐Distance and Ka/Ks)

**FIGURE 4:**
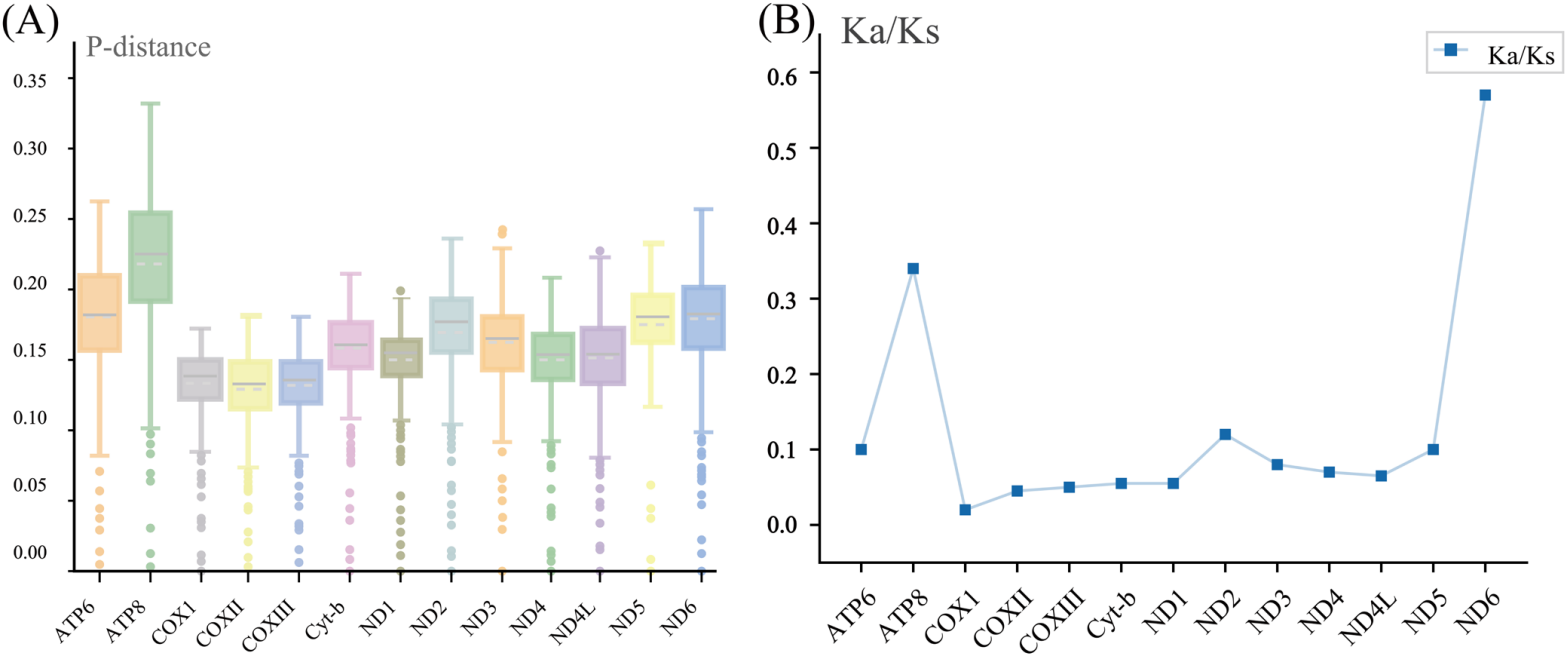
Evolutionary divergence and selective constraints of the 13 mitochondrial protein‐coding genes among crotaline species. (A) Mean p‐distance values calculated for each protein‐coding gene, indicating the relative level of nucleotide divergence among species. (B) Ka/Ks ratios calculated for each protein‐coding gene, indicating the strength of selective constraint; values below 1 suggest purifying selection.

To compare evolutionary divergence among mitochondrial protein‐coding genes, mean p‐distance values were calculated for each gene among the sampled crotaline species (Figure 4A). Across the 13 protein‐coding genes, p‐distance values ranged from 0.130 to 0.219. ATP8 showed the highest p‐distance value (0.219), indicating the greatest nucleotide divergence among the genes analyzed, whereas COX2 showed the lowest value (0.130), indicating relatively high sequence conservation.

Ka/Ks ratios were calculated to assess selective constraints on the 13 mitochondrial protein‐coding genes (Figure 4B). All genes showed Ka/Ks ratios below 1, suggesting that these mitochondrial genes are subject to purifying selection. COX1 had the lowest Ka/Ks value (0.0165), indicating the strongest functional constraint, whereas ND6 had the highest value (0.5704), suggesting relatively relaxed selective constraint compared with the other protein‐coding genes.

### 3.4 Ribosomal RNA Genes and Transfer RNA Genes

The mitochondrial genome of *O. makazayazaya* contained the typical rRNA gene cluster consisting of 12S rRNA and 16S rRNA, which were separated by tRNA‐Val. The 12S rRNA gene was 913 bp in length, and the 16S rRNA gene was 1485 bp in length. Together, the two rRNA genes totaled 2398 bp and accounted for 13.92% of the complete mitogenome (Table 2).

A total of 22 tRNA genes were identified, with a combined length of 1427 bp, accounting for 8.28% of the complete mitogenome. The tRNA genes ranged from 57 bp to 73 bp in length. tRNA‐Leu1 and tRNA‐Asn were the longest, each with a length of 73 bp, whereas tRNA‐Ser2 was the shortest at 57 bp. Fourteen tRNAs were encoded on the heavy strand, and eight were encoded on the light strand (Table 2). Two tRNA‐Ser genes and two tRNA‐Leu genes were identified in the mitogenome. Secondary‐structure prediction indicated that all tRNAs could be folded into typical cloverleaf structures except tRNA‐Ser2, which lacked the dihydrouridine arm and formed a simplified loop structure. This feature is commonly observed in metazoan mitochondrial tRNA‐Ser genes.

### 3.5 Phylogenetic Analyses

**FIGURE 5:**
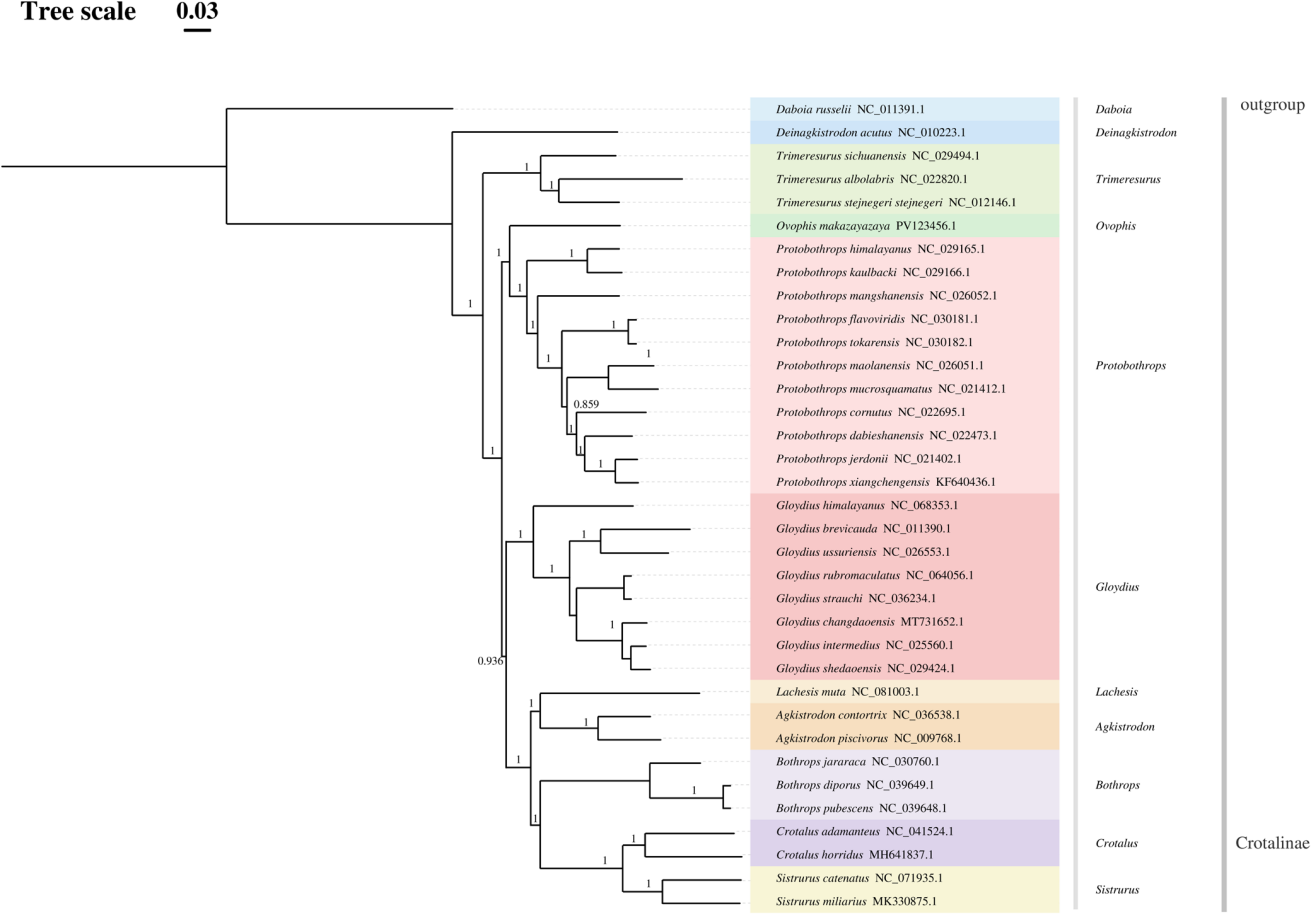
Bayesian inference phylogenetic tree inferred from the concatenated nucleotide sequences of 13 mitochondrial protein‐coding genes. Numbers at nodes indicate posterior probabilities.

**FIGURE 6:**
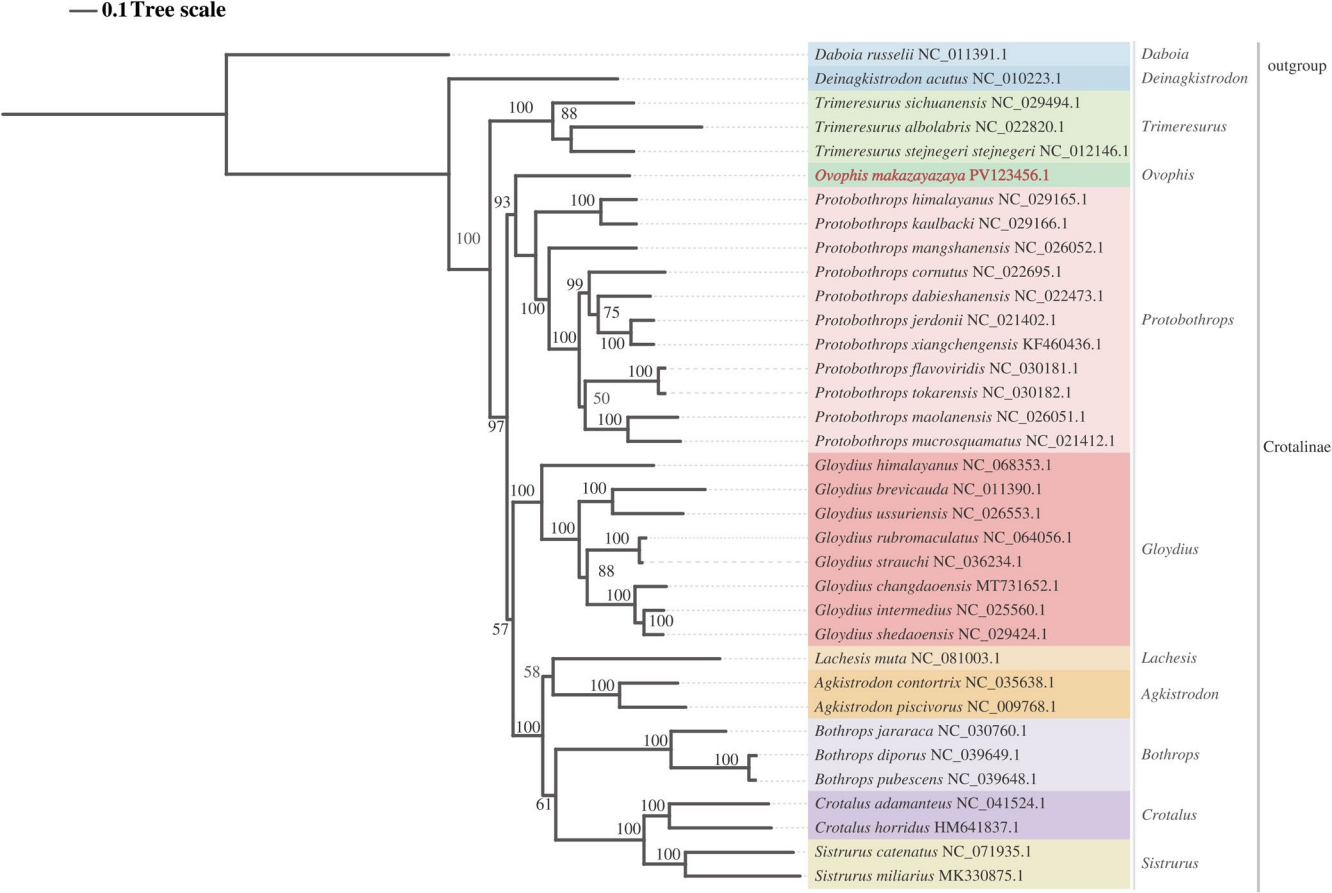
Maximum‐likelihood phylogenetic tree inferred from the concatenated nucleotide sequences of 13 mitochondrial protein‐coding genes. Numbers at nodes indicate bootstrap support values.

Phylogenetic analysis was performed based on the 13 protein‐coding gene (PCG) sequences of 33 Crotalinae species retrieved from GenBank, integrated with the newly sequenced *O. makazayazaya* (PV608488) from this study, using *Daboia russelii* (NC_011391.1) as an outgroup (Table 1). Bayesian inference (BI) and maximum likelihood (ML) analyses recovered largely congruent topologies, and most nodes were strongly supported (Figures 5 and 6). *O. makazayazaya* was nested within Crotalinae in both trees. Its placement was consistent between BI and ML, with support values of PP and BS for the node subtending *O. makazayazaya* .

## 4 Discussion

As in other crotaline pitvipers, the mitochondrial genome of *O. makazayazaya* is a typical double‐stranded, closed circular molecule (Wolstenholme 1992; Boore 1999). Its total length of 17,230 bp falls within the range commonly reported for pitvipers and includes both coding and non‐coding regions (Kumazawa et al. 1996). The genome encodes 13 protein‐coding genes involved in oxidative phosphorylation, 22 tRNAs, and two rRNAs, reflecting the conserved vertebrate mitochondrial gene complement (Anderson et al. 1981; Boore 1999). The overall base composition shows clear A + T enrichment, with an A + T content of 57.2%. Such compositional bias is widespread in animal mitochondrial genomes and is often associated with asymmetric replication, strand‐specific mutation pressure, and functional constraints on mitochondrial genes (Reyes et al. 1998; Castellana et al. 2011). The positive AT‐skew and negative GC‐skew observed in *O. makazayazaya* further indicate unequal nucleotide composition between complementary strands, which may contribute to lineage‐specific patterns of mitochondrial sequence evolution.

The *O. makazayazaya* mitogenome contains several overlapping gene regions and intergenic spacers, reflecting the compact but structurally variable nature of mitochondrial genomes. Gene overlap is common in animal mitogenomes and may help maximize genetic information within a limited genome size (Anderson et al. 1981; Boore 1999). The 1027 bp non‐coding region between tRNA‐Pro and tRNA‐Leu1 is particularly noteworthy. Large non‐coding spacers in snake mitogenomes may represent lineage‐specific structural variation, remnants of duplicated regions, or control‐region‐like sequences. However, because the function of this region was not experimentally tested in the present study, we conservatively interpret it as a large intergenic spacer based on the current annotation. Further comparative analyses with additional *Ovophis mitogenomes* will be needed to determine whether this region is unique to *O. makazayazaya* or shared among closely related lineages.

Start‐ and stop‐codon usage in *O. makazayazaya* is broadly consistent with patterns commonly reported for reptilian and other vertebrate mitogenomes (Wolstenholme 1992; Boore 1999). Most protein‐coding genes initiate with ATG, whereas ND2, COX1, and ND3 use alternative start codons, indicating flexibility in mitochondrial translation initiation. Several genes terminate with incomplete T stop codons, which are typically completed by post‐transcriptional polyadenylation (Anderson et al. 1981; Ojala et al. 1981). The RSCU analysis showed heterogeneous synonymous codon usage among protein‐coding genes, with CUA showing the highest RSCU value and ACG showing the lowest value. This codon usage pattern is likely shaped by nucleotide composition, mutational bias, and functional constraints acting on mitochondrial genes.

Comparative analyses across sampled crotaline species showed that all 13 mitochondrial protein‐coding genes had Ka/Ks ratios below 1, consistent with pervasive purifying selection on mitochondrial proteins (Hurst 2002; Meiklejohn et al. 2007; Castellana et al. 2011). This result is expected because mitochondrial protein‐coding genes are essential components of oxidative phosphorylation, and nonsynonymous substitutions may directly affect energy metabolism. COX1 showed the lowest Ka/Ks value, suggesting strong functional constraint and high sequence conservation. In contrast, ND6 showed the highest Ka/Ks value, suggesting relatively relaxed selective constraint compared with other mitochondrial genes. The p‐distance analysis also revealed gene‐specific variation in nucleotide divergence, with ATP8 showing the highest divergence and COX2 showing the lowest divergence. These differences indicate that mitochondrial genes evolve at different rates and may contribute differently to phylogenetic reconstruction and species‐level comparisons.

The rRNA and tRNA genes of *O. makazayazaya* show conserved features typical of vertebrate mitochondrial genomes. The 12S rRNA and 16S rRNA genes are separated by tRNA‐Val, which is the standard arrangement in vertebrate mitogenomes (Wolstenholme 1992; Boore 1999). The predicted rRNA secondary structures provide additional support for the annotation of these two rRNA genes. Although no highly unusual rRNA structure was detected, the visualization of 12S rRNA and 16S rRNA structures improves the transparency of the annotation and provides useful comparative information for future mitogenomic studies of Crotalinae.

Most tRNAs identified in *O. makazayazaya* were predicted to fold into typical cloverleaf secondary structures, supporting the reliability of tRNA annotation. The main exception was tRNA‐Ser2, which lacked the dihydrouridine arm and formed a simplified loop. Loss of the DHU arm in mitochondrial tRNA‐Ser is widespread among metazoans and is generally considered compatible with the mitochondrial translation system (Wolstenholme 1992; Boore 1999). Therefore, although the tRNA structures do not represent a novel or highly unusual pattern, they provide direct structural evidence for RNA‐gene annotation and support comparative characterization of the *O. makazayazaya* mitogenome.

Phylogenetic analyses based on the concatenated mitochondrial protein‐coding genes recovered *O. makazayazaya* within Crotalinae and placed it close to Protobothrops in both Bayesian inference and maximum‐likelihood analyses. This result is broadly consistent with previous molecular studies showing close relationships among Asian pitviper lineages, including Ovophis and Protobothrops (Malhotra and Thorpe 2000; Malhotra et al. 2011; Alencar et al. 2016; Zeng et al. 2023). However, the present phylogenetic analysis should be interpreted with caution. Because only one Ovophis species was included in the mitogenomic dataset, the analysis cannot directly test the monophyly of Ovophis. Instead, it provides evidence for the mitochondrial phylogenetic placement of *O. makazayazaya* within Crotalinae.

Another limitation is that the present phylogeny is based exclusively on mitochondrial protein‐coding genes. Mitochondrial genomes are useful for resolving maternal‐lineage relationships and for comparative mitogenomic analyses, but they represent a single maternally inherited marker system. Mitochondrial phylogenies may be affected by introgression, incomplete lineage sorting, or mito‐nuclear discordance (Meiklejohn et al. 2007; Castellana et al. 2011). Therefore, future studies should include denser taxon and population sampling within Ovophis, additional complete mitogenomes, nuclear genomic markers, and coalescent‐based analytical frameworks to clarify species boundaries, historical diversification, and genus‐level relationships within Ovophis and related Asian pitvipers.

Overall, the complete mitochondrial genome of *O. makazayazaya* reported here expands the available genomic resources for Crotalinae. The mitogenome shows conserved vertebrate mitochondrial organization, clear A + T bias, heterogeneous codon usage, conserved RNA‐gene structures, and strong purifying selection across protein‐coding genes. Together with the phylogenetic analyses, these results provide a useful reference for future comparative studies of mitochondrial genome evolution and phylogenetic relationships among crotaline snakes.

## Author Contributions

**Yuheng Jiang:** conceptualization (equal), data curation (equal), formal analysis (equal), investigation (equal), methodology (equal), resources (equal), software (equal), supervision (equal), validation (equal), visualization (equal), writing – original draft (equal), writing – review and editing (equal). **Yuhao Nie:** conceptualization (equal), data curation (equal), formal analysis (lead), funding acquisition (lead), investigation (equal), methodology (lead), project administration (lead), resources (equal), software (equal), supervision (lead), validation (equal), visualization (lead), writing – original draft (lead), writing – review and editing (lead). **Zhiqiang Ge:** conceptualization (equal), data curation (equal), software (equal), validation (equal).

## Funding

The authors have nothing to report. The study was self‐funded by the corresponding author, Yuhao Nie.

## Conflicts of Interest

The authors declare no conflicts of interest.

## Data Availability

The complete mitochondrial genome sequence has been deposited in GenBank under accession number PV608488. The raw sequencing reads have been deposited in the NCBI Sequence Read Archive under BioProject accession number PRJNA1467230.
